# Graphs in molecular biology

**DOI:** 10.1186/1471-2105-8-S6-S8

**Published:** 2007-09-27

**Authors:** Wolfgang Huber, Vincent J Carey, Li Long, Seth Falcon, Robert Gentleman

**Affiliations:** 1 European Bioinformatics Institute, European Molecular Biology Laboratory, Cambridge CB10 1SD, UK; 2 Channing Laboratory, Brigham and Women's Hospital, 75 Francis Street, Boston MA 02115, USA; 3 Vital-IT Center, Swiss Institute of Bioinformatics, 1015 Lausanne, Switzerland; 4 Fred Hutchinson Cancer Research Center, Computational Biology Group, 1100 Fairview Avenue North – M2-B876, P.O. Box 19024, Seattle WA 98109-1024, USA

## Abstract

Graph theoretical concepts are useful for the description and analysis of interactions and relationships in biological systems. We give a brief introduction into some of the concepts and their areas of application in molecular biology. We discuss software that is available through the Bioconductor project and present a simple example application to the integration of a protein-protein interaction and a co-expression network.

## Introduction

Molecular biology is concerned with enumerating and characterizing all the building blocks of living systems, as well as with their relationships, how the properties and the activity of one element affects those of another. For example, certain proteins have the capability of binding to particular regions of a cell's DNA, thereby activating or inhibiting the transcription of messenger RNA that codes for another protein, or even for that protein itself. Many proteins have the capability of binding to other proteins, forming a complex that can perform actions that none of the individual constituent proteins would be able to do. There are thousands, perhaps millions of different types and states of proteins in a living organism, and the number of possible interactions between them is enormous. The language of graph theory offers a mathematical abstraction for the description of such relationships. The beauty and usefulness of this abstraction is that it allows to develop concepts and tools independent of the concrete application. Many scientists and engineers are familiar with the benefits of abstraction that lie in linear algebra, calculus or probability theory; the goal of this article is to demonstrate some of the scope and power of the theory of graphs for the biology of gene regulation.

A graph consists of a set of nodes and a set of edges that connect the nodes. The nodes are the entities of interest and the edges represent relationships between the entities. For example, the entities may be a set of proteins in a cell, and the relationship modeled may be the existence of a physical interaction between two proteins. A graph is specified by the set of nodes *V *the set of edges *E*. Each element of *E *contains a pair of elements of *V*. Edges can be assigned weights, directions, and types. Sometimes, specialized forms of graphs such as multigraphs, bipartite graphs and hypergraphs [1,2] are useful.

We will use the terms *graph *and *network *interchangeably, the former stressing the mathematical concept, the latter the applications.

## Applications

Graphs play roles in three complementary areas. First, graphs provide a data structure for *knowledge representation*. Examples include regulatory, signal transduction, or metabolic networks that are represented in graph form. This might be either in the informal way of the familiar bubbles and arrows cartoons of molecular biology text books, or more formally in knowledge databases such as Reactome [3]. Graphs are also used for knowledge representation in the Gene Ontology (GO) [4], and bipartite graphs between biological concepts and scientific papers that are written about them [5] are another form of knowledge representation.

A second application of graphs to molecular biology is to model measured data. Many types of molecular biological experiments produce data that convey relationships between molecules. For example, in a Yeast-Two-Hybrid screen, the data is the observation that a pair of proteins worked together to create a transcription initiation complex. In a chromatin immuno-precipitation microarray experiment (ChIP-chip), the data is the strength of the binding of the pulled down protein to the queried DNA regions, which themselves may be linked to one or several genes whose transcription is regulated through them.

A further role for graphs is in *statistical modeling*. For example, one might want to fit a model that describes which sets of proteins can assemble together to form a protein complex, given some data consisting of (usually imperfect and incomplete) observations of pairwise interactions or of the co-precipitation of proteins [6]. Different models might apply to fit the data, and the usual questions of model fitting and discrimination and of hypothesis testing arise. Another example is the question whether interacting proteins are also transcriptionally co-regulated. This might be answered by looking at the respective networks, the protein-protein interaction graph and the co-expression graph, and testing whether and how the overlap of these graphs is more than would be expected by chance [7].

Graphical models [8] can also be used to model complicated multivariate probability distributions with a limited number of parameters. Nodes in a graphical model represent random variables, and the lack of an arc between nodes represents an assumption of conditional independence. In an undirected graphical model (sometimes this is also called a Markov random field), two (sets of) nodes *U *and *V *are conditionally independent given a third set, *W*, if all paths between the nodes in *U *and *V *are separated by a node in *W*. In a directed graphical model (sometimes this is also called a Bayesian network), a node is independent of its ancestors given its parents, where the ancestor/parent relationship is with respect to some fixed topological ordering of the nodes.

## Definitions

The presentation and notation here is based on that used in [9]. The treatment is not comprehensive, and we refer to more complete references such as [1,9,10].

A graph is specified by the set of nodes (the term *vertex *is also sometimes used) *V *and the set of edges *E*. Each element of *E *contains a pair *u*, *v *of elements of *V*. It is allowed that *u *= *v*, in which case one also speaks of a *self-loop*. The relationships modeled by the edges may be dichotomous (the edge is there or it is not there) or we may consider a more general interpretation of *E *as a two-place function *f *: *V *× *V *→ *F *with discrete or continuous range *F*. If *F *⊂ ℝ, then the value *f*(*u*, *v*) is called the weight of the edge from *u *to *v*. *F *can also extend over different discrete categories, for example, a graph with genes as nodes can simultaneously model the homology between genes and their co-citation in the medical literature.

In some cases, such as in transcription factor networks, the relationships between the nodes in the graph are directed. A graph can simultaneously contain directed and undirected relationships.

An edge is said to be *incident at *a node if the node is an endpoint for the edge. A *proper edge *is an edge that is not a self-loop, and a *multi-edge *is a set of two or more edges that have the same endpoints. A *directed edge *is an edge where one endpoint is designated the *head *and the other the *tail*. Directed edges join the tail node to the head node but not vice versa. A *directed graph*, or *digraph*, is a graph where all edges are directed. The *underlying *graph of a digraph is the graph that results from making all directed edges undirected edges.

Two nodes are said to be *adjacent *if they are joined by an edge. Two edges are adjacent if they are joined by a node. The *adjacency matrix *of a graph is a square matrix *A *whose rows and columns correspond to the nodes and whose element *A*_*ij *_denote the presence (and possibly, weight or type) of an edge from node *i *to *j*. For undirected graphs, the adjacency matrix is symmetric.

The *degree *of node *v *is denoted deg(*v*) and is equal to the number of proper edges incident at *v *plus twice the number of *v*'s self-loops. For directed graphs we define *in-degree *to be the number of edges directed at the node and *out-degree *to be the number of edges that go out from the node. A *complete graph *is a graph such that every pair of nodes is joined by an edge.

**Figure 1:**
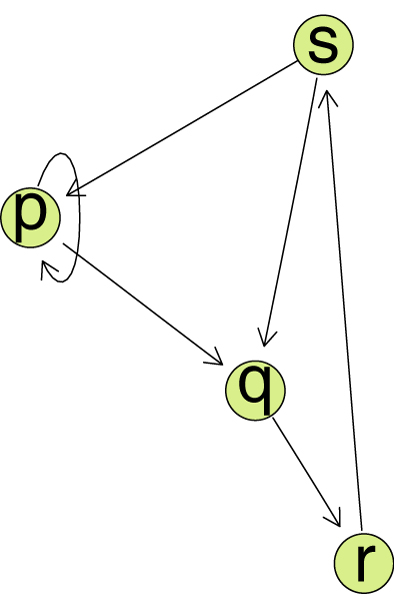
**A simple graph**. An example for a simple directed graph.

In Figure 1, node p has a self-loop, and there is no edge between nodes p and r. The other edges are all directed, as there are arrowheads only on one end.

A *walk *from node *v*_0 _to node *v*_*n *_is an alternating sequence of nodes and edges,

*W *= ⟨*v*_0_, *e*_1_, *v*_1_,⋯, *v*_*n*-1_, *e*_*n*_, *v*_*n*_⟩

such that the endpoints of *e*_*i *_are *v*_*i*-1 _and *v*_*i *_for *i *= 1,..., *n*. In a digraph we refer to the analogous structure as a directed walk. The length of a walk when no edge weights are defined is the number of edges traversed. If edge weights are defined, the length will be computed by summing the edge weights. A walk is *closed *if *v*_0 _= *v*_*n*_.

A node *v *is said to be reachable from node *u *if there is a walk from *u *to *v*. A graph is said to be *connected *if there is a walk between every pair of nodes in the graph. A digraph is said to be *weakly connected *if its underlying graph is connected. Two nodes *w *and *z *in a digraph are said to be mutually reachable if there is a directed walk from *w *to *z *and a directed walk from *z *to *w*. A digraph is said to be *strongly connected *if every pair of nodes in the digraph are mutually reachable.

The *distance *between two nodes *u *and *v *is the length of the shortest walk containing them. For a digraph the directed distance is the length of the shortest directed walk. Note that the distance function so defined for digraphs may not be symmetric in its arguments. We define a *trail *to be a walk with no repeated edges and a *path *to be a walk with no repeated nodes, except possibly the first and last. A non-trivial closed path is called a *cycle*.

For a graph *G *= (*V*, *E*) the *connectivity *is defined to be the minimum number of edges whose removal results in a disconnected graph. This number is denoted *k*(*G*). If *k*(*G*) = *l*, then *G *is said to be *l*-connected. A *cut *in *G *is a set of edges whose removal disconnects the graph. A minimum cut is a cut with the minimum number of edges. If *C *is a minimum cut of a non-trivial graph *G*, then |*C*| = *k*(*G*). The connectivity of the graph in Figure 1 is 2.

**Figure 2:**
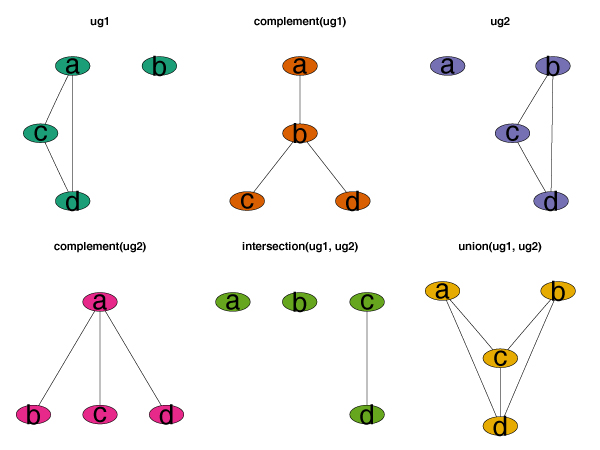
**Set operations on graphs**. Set operations on two undirected graphs ug1 and ug1.

Connectivity properties can also be described in terms of nodes. Sometimes there is interest in those nodes whose deletion from a connected graph *G *results in a disconnected graph. A *cut-set *is a node set *U *such that *G*\*U *has more components (defined below) than *G*.

A *subgraph *of *G *= (*V*, *E*) is a graph *H *= (*W*, *F*) where *W *is a subset of *V*, and *F *is a subset of *E*, and all edges in *F *have their endpoints in *W*. An *induced subgraph *is a subgraph that is defined in terms of a node set *W *⊂ *V *and contains all edges from *E *that have both endpoints in *S*. If *G *is a directed graph, then so are all subgraphs. Subgraphs can also be induced by edge sets in an analogous manner.

A *clique *is a subset of the nodes in *V *such that every pair of nodes in the subset is joined by an edge. If the clique is not a proper subset of any other clique, then we call it a *maximal clique*. A node adjacent to a node *v *is said to be a neighbor of *v*. A *component *of a graph *G *is a maximal connected subgraph. In a graph *G *we refer to the component of a node *v *as the set of nodes that are reachable from *v *and denote this *C*(*v*). Cliques are one type of cohesive subgroup, a term for sets of nodes for which there is a high degree of relatedness as demonstrated by the existence of many edges. For applications, the clique will often be too restrictive a notion of cohesive subgroup, and we will consider more general definitions below.

For the sake of simplicity, we now diverge somewhat from the standard graph theoretic terminology for concepts of graph unions and intersections. In many applications, the node and edge sets of the graphs we need to consider are subsets of a large, but limited set of nodes and edges, for example all annotated genes in a genome. We define the *union *of two graphs *G *and *H *to be the graph *F *satisfying *V*(*F*) = *V*(*G*) ⋃ *V*(*H*) and *E*(*F*) = *E*(*G*) ⋃ *E*(*H*). The *intersection *graph is defined analogously, substituting ⋂ for ⋃. The union and intersection of two graphs are themselves graphs. The *complement *of a graph *G *= (*V*, *E*) is the graph *G' *= (*V*, *E'*) where *E' *are those edges in the complete graph on *V *that are not in *E*. These concepts are presented in Figure 2.

### Cohesive subgroups

In application, the identification of maximal cliques is often of limited interest as the requirement of complete connectivity is so restrictive. When dealing with imperfect systems or with experimental data, we may need to consider more general notions of cohesive subgroups. Our description here follows that of [11]. They consider different notions of cohesive subgroups that include *n*-cliques, *k*-plexes and *λ*-sets.

An *n-clique *is a subgraph with nodes *V*_*s *_such that the distance *d*(*v*, *u*) between nodes *v *and *u *is less than or equal to *n *for all nodes *v*, *u *∈ *V*_*s*_. A 1-clique is the same as a clique.

A *k-plex *is a maximal subgraph *V*_*s *_containing *v*_*s *_nodes, in which each node is adjacent to no fewer than *v*_*s *_- *k *nodes. Let *deg*_*s*_(*u*) denote the number of edges from *u *to nodes of the subgraph *V*_*s*_. Then a *k*-plex is a subgraph *V*_*s *_such that *deg*_*s*_(*u*) ≥ *v*_*s *_- *k*, for all *u *∈ *V*_*s*_, and such that there is no node *w *in *V*\*V*_*s *_such that *deg*_*s*_(*w*) ≥ *v*_*s *_- *k*. A 1-plex is a maximal clique. For valued relationships, the requirement may be changed to require the existence of edges with value greater than *δ*.

One way to view this definition is that we are allowing up to *k *false negative edges per node. False positive edges, if infrequent, are unlikely to cause problems, because the probability that all nodes within a subgraph have a false positive edge to the same node tends to be negligible. There are exceptions, however, and in some cases the experimental technology being used may induce correlated false positive, or false negative, edges.

A *k-core *is defined similarly to a *k*-plex, with the main difference being that for a *k*-core, the minimum number of edges that must exist is specified, rather than the maximum number that can be missing.

*λ-sets*: another way to think of a cohesive subgroup is as a set of nodes that are more related to each other than they are to the other nodes. When viewed in this manner, one might look for regions of the graph in which the concentration of edges between nodes in that region is larger than the concentration of edges from that region to the rest of the graph. These ideas have been embodied in the notions of *λ*-sets [12]. Let *λ*(*w*, *u*) denote the minimum number of edges that must be removed so that there is no path between nodes *w *and *u*. For any graph *G *= (*V*, *E*), a set of nodes *W *⊂ *V *is a *λ*-set if for all *u*, *v*, *w *∈ *W *and *l *∈ *V*\*W λ*(*u*, *v*) ≥ *λ*(*w*, *l*). Borgatti et al. note that the members of a *λ*-set do not need to be adjacent [12]. They can be quite distant from each other.

### Distances

The length of paths between nodes in a graph can be used to induce a distance between nodes. In many cases, the shortest path will be used, but other alternatives may be appropriate for applications. If the graph has weighted edges, then these can easily be accommodated. Multi-graphs (graphs with multiple types of edges) can have different distances determined by the different types of edges. Other notions of distance, such as the number of paths that exist between two points [13], or the number of edge-cuts required to separate two nodes, can also be used.

For example, the Gene Ontology [4] is represented by three different directed acyclic graphs, for "biological process", "cellular process" and "molecular function". Each of the three graphs has a root, and the three roots may be considered to have one overall common root node. Various methods for assessing similarity based on GO have been used, among these [14]: (i) the similarity between subgraph *g*_*i *_and subgraph *g*_*j*_, *s*(*g*_*i*_, *g*_*j*_) is computed as the length of the shortest shared path to the root node, and (ii) the similarity between subgraph *g*_*i *_and subgraph *g*_*j*_, *s*(*g*_*i*_, *g*_*j*_) is computed as |*g*_*i *_⋂ *g*_*j*_| divided by |*g*_*i *_⋃ *g*_*j*_|. We note though that the relations in the GO graph are not designed to imply distances between the terms.

Once a decision has been made about a distance measure for objects organized in a graph, standard tools for cluster analysis or multidimensional scaling can be applied to the inter-object distances. Naturally, the choice of the distance measure is essential for outcome of the analysis, and the choice should not be driven by mathematical or computational convenience, but rather by a good understanding of the biological question.

## Special types of graphs

There are special types of graphs that deserve attention because they play important roles in applications. The main ones are bipartite graphs, hypergraphs, and directed acyclic graphs (DAGs).

### Bipartite Graphs

If the nodes of a graph *G *= (*V*, *E*) can be partitioned into two sets *U *and *W *such that every edge in *E *is an undirected relationship between one node in *U *and one node in *W*, then *G *is said to be a *bipartite graph*. Note that there can be no edges between the elements of *U *or between the elements of *W*. Thus relationships between nodes in *U *are mediated through the nodes in *W *and vice versa.

Two graphs called *one mode graphs *can be derived from a bipartite graph. If *U *and *W *are the node partitions of a bipartite graph *G*, then the edges in the one mode graph on *U *(resp. *W*) are determined by whether or not the two nodes both have edges in *G *to a common element of *W *(resp. *U*). If *A *is the |*U*| × |*W*| adjacency matrix of the bipartite graph, then the one mode graph for the node set *U *can be obtained by *A *⊗ *A*^*t *^and the one mode graph for *W *by *A*^*t *^⊗ *A*. Here, ⊗ represents matrix multiplication under the Boolean algebra 0 + 0 = 0 × 0 = 1 × 0 = 0 × 1 = 0 and 1 + 0 = 0 + 1 = 1 + 1 = 1 × 1 = 1.

The *mode *of a network is the number of partitions of the node set determined by some general node property. For example, a two-mode network can be used to describe the relationships between transcription factors and target genes, or between proteins and protein complexes. In each of these cases, the node set can be partitioned by node type. Two-mode graphs are often referred to as *affiliation networks*.

In social network analysis, the two types of nodes are often referred to as *actors *and *events*. Among the basic ideas that are represented by such graphs is the concept that relationships between actors are mediated by the events that they attend (in that application domain, for example, the clubs or social groups that they belong to).

It is worth noting that *adjacency *in the one-mode graphs means that both nodes have an edge to (at least one) common node in the other node set. However, accessibility is less easy to interpret. Two nodes that are *accessible*, but not adjacent have a connection or relationship that is less direct – they are connected by a sequence of related actors and events but do not themselves share memberships directly.

### Hypergraphs

Hypergraphs are closely related to bipartite graphs [1,2]. Hypergraphs generalize the graph concept, allowing for the specification of relationships that are one to many and many to many.

A *hypergraph G *is defined as a pair (*V*, *E*), where *V *is a set of nodes, and *E *is a set of hyperedges. Each hyperedge is a set of vertices, *E*_*i *_= {*u*, *v*,...} ⊂ *V*.

The hyperedges in a *directed hypergraph *are directed, and each hyperedge is an ordered pair, *E*_*i *_= (*X*, *Y*), of disjoint subsets of nodes; *X *is the tail of *E*_*i *_while *Y *is the head. A *path P *from a node *u *to a node *v *is a sequence (*V*_0 _≡ *u*, *E*_1_, *V*_1_,..., *E*_*n*_, *V*_*n *_≡ *v*) of alternating nodes and hyperedges where each hyperedge *E*_*i *_is distinct, and for *i *∈ {1,..., *n*}, *V*_*i*-1 _= tail(*E*_*i*_) and *V*_*i *_= head(*E*_*i*_).

### Directed acyclic graphs

An important class of directed graphs are the *directed acyclic graphs *(DAGs), which are simply directed graphs with no cycles. We note that a *tree *is a connected graph that has no cycles. DAGs have found many uses in statistics. They form the basis for graphical models [8,15]. They also play important roles in structuring concepts, both GO and MeSH are represented as DAGs. In the Section *Case Strudy*, we demonstrate some of their uses in different specific problems.

## Uncertainty and missing edges

Using graphs as models for data analysis and data representation poses a number of challenges. In many cases, the reported graphs are imperfect.

While the presence of an edge between two nodes has usually a well-defined interpretation, for non-edges the interpretation is often less clear. We can distinguish between two cases: the existence of the edge was tested and not found, or it was never tested in the first place. Both cases are usually reported by the absence of an edge, but their interpretation is quite different.

The error rates in binary data are often described by the concepts of false positives and negatives, but in many applications we will need to address the following three categories:

**false positives **– relationships that appear in the experimental data, but are not real;

**false negatives **– relationships that are real and were probed experimentally, but were erroneously not detected; and

**untested relationships **– where no measurement was attempted and hence no information is available.

In order to make appropriate use of the data, we will need to keep these issues in mind as we explore the resultant graphs. Uncertainty is usually not part of a purely mathematical approach to graph theory, but it cannot be ignored in the context of experimental data. Uncertainty affects how we use and think about graphs or networks. Uncertainty of relationships being modeled also impacts the design of software, the choice of algorithms, and the interpretation of the output.

Particular attention is due to the fact that the three sources of error mentioned above do often not occur "randomly", but may be associated with properties of the nodes. For example, more research has been directed towards genes that are known to be implicated in human diseases, hence it should come as no surprise that literature-based interaction networks are more dense, and may indeed contain more false positives and less untested relationships in regions around these genes and than around less popular genes.

## Computational aspects

### Representation

An abstract graph can be represented for computational purposes in many different ways. Among the common representations are

**node and edge list **– a list whose elements correspond to the nodes in the graph, and each element consists of two objects: the name of a node, and the list of other nodes to which it is connected.

**adjacency matrices **– a square matrix whose elements can be Boolean, real-valued or categorical variables and denote the existence, weight or type of an edge.

**from-to matrices **– a matrix with two or more columns, each row contains start and end nodes of an edge and possibly weights, types, etc.

For bipartite graphs with node sets *U*, *W*, the adjacency matrix simplifies into a |*U*| × |*W*| matrix *A*.

The representation used for a graph can have a profound effect on the running time of algorithms that are applied to it. It is advisable to make timing comparisons on different representations before committing to a particular one. The most appropriate or efficient strategy for representing the graph will depend on many factors such as the size of the graph and the types of operations that are going to be applied to it. The *graph *package of the Bioconductor software system offers methods to translate between representations, a process sometimes referred to as "coercion." We also note that there is a close relationship between the node and edge list representation and that of sparse matrices.

### Algorithms

There are many existing, well-tested and high-quality implementations of graph algorithms. It is inefficient and often more error-prone to reimplement algorithms for which good implementations already exist. Bioconductor provides interfaces to many of the algorithms coded in the open source Boost graph library [16].

However, good implementations for many of the algorithms required in bioinformatics are still needed. Algorithms adapted to deal with incompleteness and uncertainty are of particular interest. For example, Scholtens and Gentleman [6] developed a special form of *clique *that is appropriate for protein complex data where different forms of uncertainty are prevalent. For hypergraphs, Krishnamurthy et al. describe an extension of depth first search [17], and Klamt and Gilles developed an analog of the mincut algorithm for biochemical reaction networks [18].

### Software from the Bioconductor project

Bioconductor is an open source and open development software project for the analysis and comprehension of genomic data [19]. It provides a large collection of software for the analysis of functional genomics data and among that, software for working with graphs. The software is organized into functions and packages. Functions are the basic unit of functionality and documentation. Packages contains sets of related functions for a particular domain, and they are the basic unit of authoring, versioning, dependency, distribution and deployment.

Among the graphs-related packages, it is worth differentiating between packages that are mainly infrastructure (sets of tools that can be used to create other pieces of software) and packages that are designed to provide an end-user application. The packages *graph*, *RBGL *and *Rgraphviz *are infrastructure packages. Basic data structure definitions and methods are provided in the Bioconductor package *graph*. The package *RBGL *is currently the primary source of software for graph algorithms. Package *Rgraphviz *provides graph visualization. Software developers may use these packages to construct tools aimed at specific applications areas, such as the *GOstats *or *apComplex *packages.

The *graph *package is entirely a creation of the Bioconductor core. The package *RBGL *is an interface to the Boost Graph Library [16], a C++ library devoted to portable implementation of Standard Template Library (STL) concepts for graph computations. In addition further algorithms, for example the cohesive subgroup algorithms used in the example below, were implemented by the authors (L.L.). *Rgraphviz *is an interface to Graphviz [20], a C/C++ library devoted to layout and visualization of graphs encountered in telecommunications research. We greatly appreciate the fact that the Boost and Graphviz groups have produced high-quality software with sufficiently open licenses to meet our requirements.

## Case study: using graphs for comparing transcription and interaction data

As a very simple example, we demonstrate how graph concepts can be used to do an analysis that relates gene expression data to protein interaction data.

Proteins that form a functional complex need to be expressed concurrently, hence we expect that something can be learned from comparing co-expression and protein complex co-membership. In particular, we consider the question is whether genes in a protein complex are more likely to have a similar pattern of gene expression than genes in different complexes.

The analysis that we demonstrate in the following was reported by Balasubramanian et al. [14] and is based on the work of Geone et al. [7]. Balasubramanian et al. used two graphs defined on a common set of nodes: the genes present in yeast. The relationship represented by the edges in the first graph is co-membership in a cluster of correlated expression, while the edges in the second graph represent co-membership in a protein complex.

**Figure 3:**
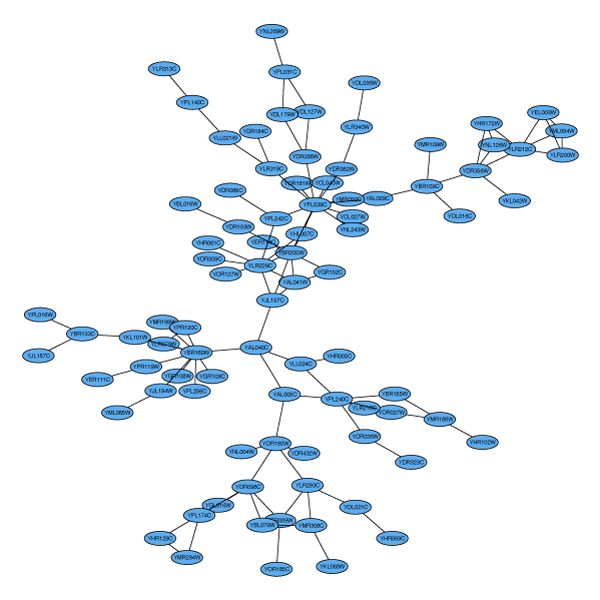
**The largest connected component of the PPI graph**. Layout of the connected component sG1 of the protein-protein interaction graph litG.

**Figure 4:**
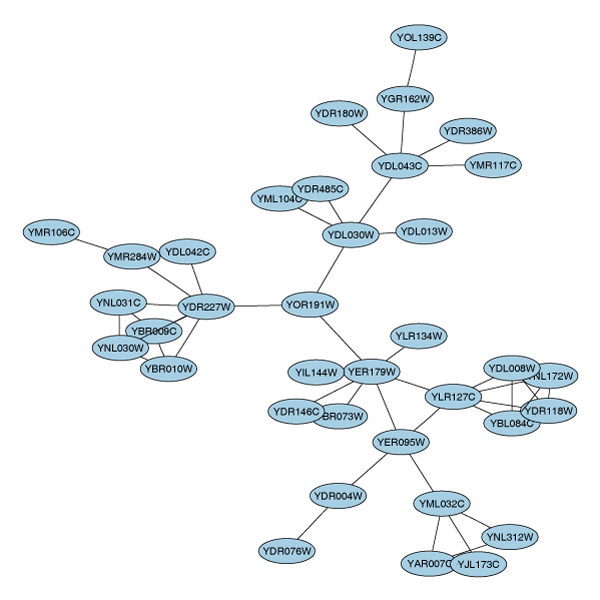
**The second-largest connected component of the PPI graph**. Layout of the connected component sG2 of the protein-protein interaction graph litG.

**Figure 5:**
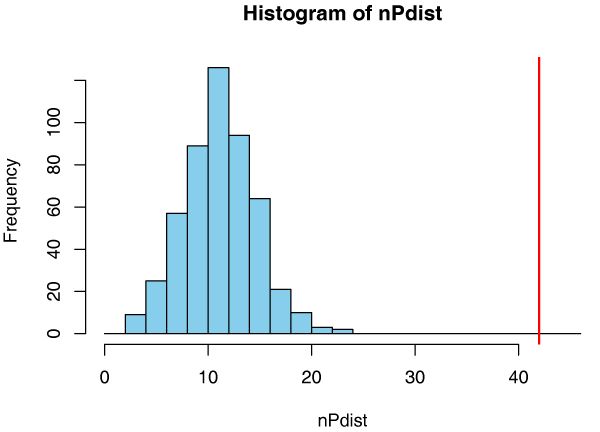
**Statistical significance of the overlap between PPI and co-expression graphs**. The *x*-position of the vertical line is the number of edges of the intersection graph between litG, the literature-curated protein-protein interaction graph, and clG, the cell cycle co-expression graph. The histogram shows of the permutation distribution obtained by random node label permutations. We conclude that the observed overlap is larger than what would be expected by chance.

**Figure 6:**
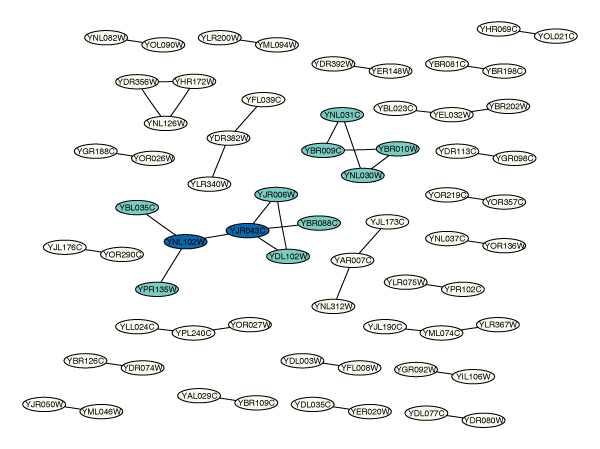
**2-cliques in the overlap graph**. Layout of the overlap graph commomnG. There are three 2-cliques, each of size 4, marked by node color. Two nodes are part of two different 2-cliques, marked in a darker color.

**Figure 7:**
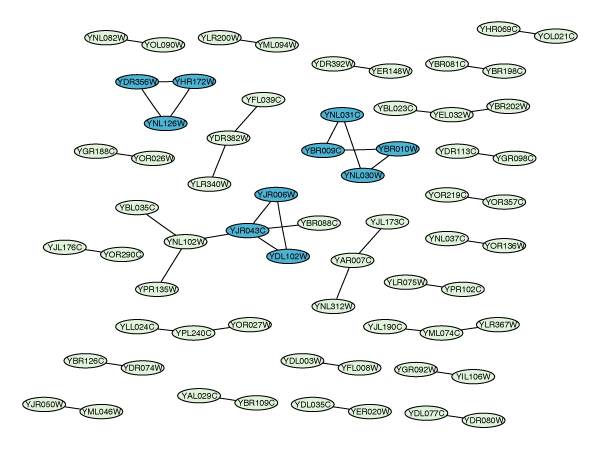
***k*-cores in the overlap graph**. Layout of the overlap graph commonG. Three 2-cores are marked by node color.

For concreteness, we will show the R programming code to perform this analysis. Figures 3, 4, 5, 6, 7 are generated from the results of these computations, and the *Sweave *source document for this article includes all the R code for analysis and graphics displays. It is available as additional file 1.

We set up the comparison by creating the two graphs as objects in the R language and counting how many edges they have in common. To see whether this number is significantly above what could be expected *by chance *if there were no relationship between protein complex co-membership and co-expression. There are some subtleties in the definition by what we mean by *by chance*, as we will discuss below.

### The Data

The R package *yeastExpData *contains the gene expression data from a yeast cell-cycle time course [21], including an assignment of the genes into co-expressed clusters in the dataframe ccyclered, and protein-protein interaction (PPI) data extracted from published papers (litG).

> library("yeastExpData")

> data("ccyclered")

> ccyclered [1:2, 1:8]



> table(ccyclered$Cluster)



> data("litG")

> litG

A graphNEL graph with undirected edges

Number of Nodes = 2885

Number of Edges = 315

The code above shows the first two rows (genes) of ccyclered, the sizes of the 30 clusters, and a summary of the *graph *object litG.

### Exploration of the PPI graph

To explore the graph litG, we can employ the functionality of the package *RBGL*. First, we find the connected components.

> library("RBGL")

> cc = connectedComp(litG)

> table(listLen(cc))



cc is a list of the connected components of litG. There are 2587 singletons (connected components of size 1), and the largest connected component has size 88. Let us plot the two largest components using the *Rgraphviz *package. We first determine the indices of the ordered components,

> ord = order(listLen(cc), decreasing = TRUE)

select the largest subgraph,

> sG1 = subGraph(cc [[ord[1]]], litG)

lay it out using the function agopen, which is an interface to the *graphviz *graph layout library, and plot it. There are many options for node color, line color and type, node shape etc., for which we refer to the vignette of the *Rgraphviz *package.

> lsG1 = agopen(sG1, layoutType = "neato", nodeAttrs = makeNodeAttrs(sG1, + fillcolor = "steelblue2"), name = "sG1")

> plot(lsG1)

The graph is shown in Figure 3. Similarly, Figure 4 shows the second-largest connected component sG2.

> sG2 = subGraph(cc [[ord[2]]], litG)

### Construction of the cluster graph

There is a specialized graph class *clusterGraph *that can be used to represent clusters. The 30 clusters of the 2885 genes in the ccyclered dataset are represented by 30 subgraphs which are fully connected within themselves and unconnected with each other.

> clusts = with(ccyclered, split(Y. name, Cluster))

> clG = new("clusterGraph", clusters = clusts)

### Statistical analysis of the graph overlap

It is now easy to determine how many pairs of genes have both a protein-protein interaction and are found in the same expression cluster. We find the intersection of the cluster-graph and the literature graph using the R function intersection.

> commonG = intersection(clG, litG)

A graphNEL graph with undirected edges

Number of Nodes = 2885

Number of Edges = 42

We find that 42 edges are in common, now we will try to determine whether this number is statistically interesting, i. e. different from what could be expected by chance. We will do this by generating a null distribution via permutation of node labels on the observed graph. The following function implements this.

> nodePerm = function(g, h, B = 500) {

+     n = nodes(g)

+     sapply(1:B, function(i) {

+        nodes(g) <- sample(n)

+        numEdges(intersection(g, h))

+     })

+ }

> nPdist = nodePerm(clG, litG)

Figure 5 shows the histogram of nPdist together with a vertical line at 42, the number of edges of the intersection graph. The largest number of common edges in the permutation distribution is 24. We conclude that the overlap between litG and clG is statistically significant. In the next section, we will do some data exploration to investigate some of the biological significance.

### Cohesive subgroups

Let us look at cohesive subgroups of the intersection graph commonG. First, we remove the singleton nodes,

> sel = names(which(degree(commonG) >= 1))

> commonG = subGraph(sel, commonG)

then we use the functions from the *RBGL *package to identify the different types of cohesive subgroups that were defined above.

> kcliq = kCliques(commonG)

> kcore = kCores(commonG)

> lambd = lambdaSets(commonG)

kcliq, the return value of kCliques is a list whose *k*-th entry is a list of all the *k*-cliques in the graph. We can get all the 2-cliques of size >= 4,

> listSel = function(x, n) x[listLen(x) >= n]

> kc2 = listSel(kcliq[[2]], 4)

> kc2

[[1]]

[1] "YBR009C" "YBR010W" "YNL030W" "YNL031C"

[[2]]

[1] "YBL035C" "YJR043C" "YNL102W" "YPR135W"

[[3]]

[1] "YBR088C" "YDL102W" "YJR006W" "YJR043C" "YNL102W"

Remember that a 2-clique is a subgraph in which the distance between each pair of nodes is ≤ 2. Any subgraph of size ≤ 3 satisfies this requirement trivially, hence we consider those with size ≥ 4. They are shown in Figure 6. Using the gene annotation data provided in the metadata package *YEAST*, we can look at the names and descriptions of the 4 genes in the second 2-clique.

> library("YEAST")

> mget(kc2[[2]], YEASTGENENAME)

> mget(kc2[[2]], YEASTDESCRIPTION)

YBL035C YJR043C YNL102W YPR135W

"POL12"   "POL32"   "POL1"       "CTF4"

YBL035C

B subunit of DNA polymerase alpha-primase complex, required for initiation of DNA replication during mitotic and premeiotic DNA synthesis; also functions in telomere capping and length regulation

YJR043C

Third subunit of DNA polymerase delta, involved in chromosomal DNA replication; required for error-prone DNA synthesis in the presence of DNA damage and processivity; interacts with Hys2p, PCNA (Pol30p), and Pol1p

YNL102W

Catalytic subunit of the DNA polymerase alpha-primase complex, required for the initiation of DNA replication during mitotic DNA synthesis and premeiotic DNA synthesis

YPR135W

Chromatin-associated protein, required for sister chromatid cohesion; interacts with DNA polymerase alpha (Pol1p) and may link DNA synthesis to sister chromatid cohesion

The first 2-clique is a duplicated pair of histone proteins:

> sapply(kc2[[1]], function(i) YEASTGENENAME [[i]])

YBR009C YBR010W YNL030W YNL031C

"HHF1" "HHT1" "HHF2" "HHT2"

A *k*-core is a subgraph in which every node is connected to at least *k *other nodes within the subgraph. The 2-cores of commonG are shown in Figure 7. lambd represents the *λ*-sets of commonG. It has 2 elements, the first one is the maximum degree *k*_max _in the graph, the second is a list of length 3 with the *λ*-sets for *k *= 0, 1, and 2, respectively.

> lambd[[1]]

[1] 2

> names(lambd[[2]])

[1] "lambda-0 sets" "lambda-1 sets" "lambda-2 sets"

> lambd[[2]] [[3]]

[[1]]

[1] "YBR009C" "YBR010W" "YNL030W" "YNL031C"

[[2]]

[1] "YDL102W" "YJR006W" "YJR043C"

[[3]]

[1] "YDR356W" "YHR172W" "YNL126W"

In this particular example, we note that the *λ*-sets for *k *= 2 are the same as the 2-cores in Figure 7, hence there is no need for an extra figure.

## Discussion

There are many ways in which graphs play a role in computational molecular biology, among these the representation and integration of experimental datasets as graphs; the interactive navigation and visualization of these large and complex datasets by a human researcher; the computation of solutions to problems such as cliques and cohesive subgroups, graph alignment, optimal paths or path-sets; the estimation of and statistical inference on an underlying ("hidden") graph from noisy observational data.

There is a substantial body of existing methodology in graph theory that is relevant to these questions, and it is a challenging and exciting task to establish the most appropriate and effective models. There is a need for theoretical development of the field, but also for software that integrates data analytic and statistical inference capabilities with methods for querying and manipulating graphs.

We have produced an approach to such an environment in Bioconductor. We made extensive use of existing software in particular from the Graphviz [20] and Boost Graph Library [16] projects, connecting them together using the R system with its powerful computational engine and elegant programming language. However, much remains to be done.

## Supplementary Material

Additional File 1 **Sweave source code of the article**. The Sweave markup of this paper, including the text in LATEX format and the program code for the example analysis in the Case Study and the generation of all figures. Click here for file
